# Association between place of dental check‐ups and work absenteeism among Japanese workers

**DOI:** 10.1002/1348-9585.12415

**Published:** 2023-06-24

**Authors:** Sayo Masuko, Takashi Zaitsu, Akiko Oshiro, Miho Ishimaru, Jun Aida

**Affiliations:** ^1^ Department of Oral Health Promotion, Graduate School of Medical and Dental Sciences Tokyo Medical and Dental University Bunkyo‐ku Japan

**Keywords:** absenteeism, dental check‐ups, dental visits, oral health, productivity loss

## Abstract

**Objectives:**

Dental check‐ups at the workplace provide the opportunity for early detection of dental diseases. Dental check‐ups during working hours could reduce the number of days of absence from work due to visits to dental clinics outside the workplace. Although health check‐ups are provided to workers in Japan, dental check‐ups is not mandatory. This study aimed to determine the association between the place of dental check‐ups and absenteeism due to visits to the dental clinic.

**Methods:**

This cross‐sectional study used data from an online self‐reported worker survey conducted for 2 weeks in March 2017. We applied linear regression analysis with robust variance to determine the association between the place of dental check‐ups and absenteeism due to dental clinic visits while adjusting for sociodemographic, health, and oral health covariates.

**Results:**

The average age of the 3930 participants was 43.3 ± 11.7 years, and 52.3% were male. The number of days of absenteeism due to dental clinic visits in the past year for those who received check‐ups only at the dental clinic and at the workplace were 0.57 ± 2.67 days and 0.21 ± 1.20 days, respectively. After adjusting for covariates, it was found that those who received dental check‐ups at the workplace had 0.35 (95% CI, 0.12–0.58) fewer days of absence than those who received dental check‐ups at the dental clinic.

**Conclusion:**

Workers who received dental check‐ups at the workplace were associated with fewer days of absence due to dental visits than those who received at the dental clinic.

## 1 INTRODUCTION

Work absenteeism due to diseases or their treatment reduces work productivity. For instance, it was found that mental and musculoskeletal disorders can lead to financial loss of $520 per person per year due to absenteeism, which was higher than the medical and pharmaceutical costs per person per year of $1165 in Japan.^1^ The estimated national diabetes cost in the U.S. was $327 billion in 2017, of which $237 billion (73%) were direct medical costs, including treatment costs attributable to diabetes, and $90 billion (27%) was due to productivity losses from work‐related absenteeism and other factors.^2^ Therefore, labor loss due to illness and its treatment is a critical problem worldwide.

Oral diseases are one of the common work absenteeism causes because of their high prevalence. Worldwide, 29.4% of the population suffered from untreated permanent dental caries.^3^ A systematic review reported that absenteeism due to oral problems accounts for 9.06%–26.7% of absenteeism due to all illnesses.^4^ According to a previous Canadian study, 35.1% of people lost time from work or school due to dental problems and treatment.^5^ At the population level, 40 million hours were lost yearly due to dental treatment and dental problems. This productivity loss is counted as an “indirect cost” of oral diseases.^6^ Globally, these “indirect costs” of oral diseases reached $187.61 billion in 2015.^7^

Dental check‐ups can prevent the progression of oral diseases.^8^, ^9^ In Japan, it is mandatory for companies to provide the facility of annual health check‐ups as a health promotion activity; however, provision of dental check‐ups is optional. Some companies conduct dental check‐ups along with annual medical check‐ups as a part of their health promotion programs. Regular dental check‐ups at the workplace can improve oral health through timely detection of oral diseases, leading to early treatment and promotion of oral health education. In addition, conducting dental check‐ups during working hours for workers can reduce the likelihood of them taking time off from work to visit the dental clinic for check‐ups. Moreover, those who receive dental check‐ups at a dental clinic outside the workplace do not necessarily have the opportunity to receive regular dental check‐ups, which may increase the likelihood of needing emergency dental care, thereby increasing absenteeism.

However, no study has evaluated the association between the place of dental check‐ups and absenteeism from work. Therefore, we aimed to determine the association between the place of dental check‐ups and work absenteeism due to dental check‐ups. We hypothesize that dental check‐ups at the workplace would be associated with less absenteeism due to dental visits compared to dental check‐ups at the dental clinic outside the workplace.

## 2 METHODS

### 2.1 Data source

This cross‐sectional study used data from an Internet survey. The survey was a service provided by Company M, the largest service provider that conducts online surveys in Japan. Equal number of males and females were recruited as participants in this cross‐sectional survey (4000 in total), belonging to each of the 11 Japanese Standard Classifications of Occupational Categories defined by the Ministry of Internal Affairs and Communications and equally including workers from all occupational categories. Part‐time and other non‐occupational workers who fell into the “unclassifiable category” were excluded from this study. The age range for monitors of Company M was age 6 years and older, and the participants recruited for this study included individuals aged 20–65 years to focus on a specific age range for workers. The self‐administered survey was conducted online for 2 weeks in March 2017. A total of 3930 participants (2057 males and 1873 females) who consented and participated in the survey were included in the analyses, as the maximum number of 4000 participants was not reached within the recruitment period.

### 2.2 Dependent variable: Number of days of absence due to dental visits in the past year

Days of absence due to dental visits was considered a dependent variable for absenteeism. Participants were asked the following question: “How many days did you visit the dentist in the past year? If you did not visit the dentist, please enter 0 days.” In response to this question, participants were asked to answer the following: (1) the number of days they missed work to go to the dentist, (2) the number of days they came to work late or left work early to go to the dentist, (3) the number of days they used holidays to go to the dentist, and (4) the number of days they went to the dentist after work or during breaks. For the analysis, the sum of days answered for (1) and (2) was used as the dependent variable.

### 2.3 Independent variables: The place of dental check‐ups

The place at which participants underwent dental check‐ups in the past year was considered the independent variable. Participants were asked a question: “Have you had regular dental check‐ups in the past year?” This question inquired about their experiences of dental check‐ups at and outside the workplace. Then, we segregated the participants into four categories: not having check‐ups, checked up at a dental clinic, checked up in the workplace, and checked up at both the dental clinic and workplace.

### 2.4 Covariates

Age, gender, personal income (<¥2 million, ¥2–4 million, ¥4–6 million, ¥6–8 million, >¥8 million, and unknown), academic background (graduated from high school, graduated from vocational school/junior college, graduated from university/master's, or doctoral course completion/others), presence of chronic disease (no, yes), Japan Standard Industrial Classification, Japan Standard Classification of Occupations, and working pattern were included as covariates. Chronic disease presence was evaluated by the question, “Do you currently have a chronic disease (diagnosed by a doctor)?” Furthermore, three oral health statuses such as self‐reported oral health, the number of teeth, and gum bleeding were obtained. These three oral health questions have been used and validated in previous studies.^10^, ^11^, ^12^ Self‐reported oral health was asked as, “How is the health of your teeth and gums?” with five choices: Excellent, Good, Fair, Poor, and Very Poor. The number of teeth was enquired using the following question: “How many teeth do you have? This number included the number of covered teeth (gold/silver teeth), post crown, and teeth with only roots remaining.” The number of teeth was divided into five categories on the basis of distribution of participants: 0–14 teeth, 15–19 teeth, 20–23 teeth, 24–27 teeth, and 28–32 teeth. The following question was used to enquire the status of gum bleeding: “Do you bleed when you brush your teeth?” Participants were given three choices: “Always,” “Occasionally,” and “Never.” Based on previous studies, we used industry classification, occupational classification, and working pattern as variables related to occupation.^13^ The occupation of participants were divided into the following 20 categories based on the Japan Standard Industrial Classification (Revised October 2013), 12th edition^14^: (a) farming and forestry; (b) fishery; (c) mining, quarrying, and gravel extraction; (d) construction; (e) manufacturing; (f) electricity, gas, heat, and water supply; (g) telecommunications; (h) postal and transportation services; (i) retail and wholesale; (j) insurance and finance; (k) goods rental and real estate business; (l) academic research, professional, and technical services; (m) accommodation and food services; (n) lifestyle‐related services and entertainment; (o) teaching and study support; (p) medical care and welfare; (q) combined services business; (r) service industry (not classified elsewhere); (s) public affairs (excluding services classified as other); and (t) nonclassifiable industries. Of these industries, (a) and (b) are considered “primary industries,” (c–e) are considered “secondary industries,” and (f–t) are considered “tertiary industries.” Participants' occupations can also be divided into the following 11 occupations according to the Japan Standard Classification of Occupations (December 2009 Statistical Standard Setting Classification)^15^: (a) administrative occupational workers (company executives, company management staff, administrative public officials, etc.); (b) professional and technical occupational workers (research workers, health care workers, teaching staff, etc.); (c) clerical workers (personnel and labor affairs, accounting, management, etc.); (d) sales workers (sales, sales floor personnel, stocking, etc.); (e) service workers (facility and equipment maintenance, customer centers, home helpers, beauticians, etc.); (f) security career employees (self‐defense officers, policemen, guards, etc.); (g) agriculture, forestry and fishing workers (landscape architects, fishermen, navigators, etc.); (h) manufacturing process workers (steelmaking maintenance control and supervision personnel, gum and plastic material production workers, etc.); (i) transportation and machine operation workers (taxis and bus drivers, conductors, etc.); (j) construction and quarrying workers (carpenters, plumbers, civil workers, etc.); and (k) transportation, cleaning and packing workers (delivery staff, cleaning staff, etc.). Among these, categories (a–d) and (e–k) were classified as “white‐collar” and “blue‐collar,” respectively.^16^ Work patterns were then classified into four groups: day shift, night shift, both day and night shift, and flexible work timings.

### 2.5 Statistical analysis

Cross‐tabulation was made to observe the difference of dental check‐ups in the past year by oral health status, age, gender, personal annual income, educational background, presence or absence of chronic illness, industrial classification, occupational classification, working pattern, and absenteeism. Linear regression with robust variance was used to assess the association between the independent variables and absenteeism. This approach was used because the coefficients of linear regression analysis are easily comprehensible, as they can be interpreted as absolute difference in number of days of absenteeism, and they are robust to violations of the normality assumption.^17^ Univariable and fully adjusted coefficients and 95% confidence intervals (CI) of the number of days of absenteeism due to dental clinic visits were calculated. The Stata/MP 17.0 was used to perform all analyses. The significance level was set to 5%.

## 3 RESULTS

The number of participants included in the analysis was 3930 and 52.3% were male with an average age of 43.3 ± 11.7 years. The minimum age was 20 years old and the maximum age was 65 years old. Table 1 shows the descriptive statistics of the participants by the place of the dental check‐ups. Among those who received dental check‐ups at the dental clinic, the mean number of days of absenteeism due to dental visit was 0.57 ± 2.67 days. On the other hand, those who did not receive dental check‐ups (0.14 ± 1.18 days), received check‐ups at the workplace (0.21 ± 1.20 days), and received check‐ups at both the dental clinic and the workplace (0.32 ± 1.29 days) had fewer days of absenteeism due to dental visits.

**TABLE 1:** Distribution of participants by the place of dental check‐ups in the past year (*n* = 3930).

	Total	Place of dental check‐ups(%)			
	*n* (%)	Not had the check‐ups	At the dental clinic	At the workplace	At both the dental clinic and the workplace
	(*n* = 3930)	(*n* = 2411)	(*n* = 1116)	(*n* = 161)	(*n* = 242)
Self‐reported oral health					
Excellent	307 (7.8)	6.4	8.8	11.2	14.9
Good	948 (24.1)	21.0	28.9	23.0	33.9
Fair	1676 (42.6)	44.1	40.8	43.5	36.4
Poor	857 (21.8)	24.3	18.4	20.5	13.6
Very Poor	142 (3.6)	4.1	3.2	1.9	1.2
Number of teeth					
0–14	183 (4.7)	4.8	3.8	6.2	6.2
15–19	279 (7.1)	7.3	6.6	5.6	8.7
20–23	919 (23.4)	23.6	21.7	26.1	27.7
24–27	921 (23.4)	23.2	23.7	22.4	25.2
28–32	1628 (41.4)	41.1	44.3	39.8	32.2
Gum bleeding					
Always	159 (4.0)	4.5	2.7	7.5	3.7
Occasionally	1607 (40.9)	42.3	38.3	41.0	38.4
None	2164 (55.1)	53.2	59.1	51.6	57.9
Age (years)					
20–29	625 (15.9)	16.7	12.4	22.4	20.2
30–39	969 (24.7)	24.2	23.8	34.8	26.4
40–49	1011 (25.7)	26.0	26.3	19.9	24.4
50–59	888 (22.6)	22.0	25.1	15.5	21.9
60–65	437 (11.1)	11.2	12.5	7.5	7.0
Gender					
Male	2057 (52.3)	57.5	42.1	52.8	47.5
Female	1873 (47.7)	42.5	57.9	47.2	52.5
Education (graduation)					
High school	1416 (36.0)	39.9	31.7	26.1	24.4
Vocational school, Junior college	839 (21.3)	20.8	24.3	14.9	17.4
University, Master's program, Doctoral program	1675 (42.6)	39.3	44.0	59.0	58.3
Personal income					
<¥2 million	577 (14.7)	15.7	14.7	6.2	9.9
¥2–4 million	1479 (37.6)	38.6	37.3	32.3	33.5
¥4–6 million	810 (20.6)	19.9	19.9	26.7	27.3
¥6–8 million	299 (7.6)	6.9	8.5	9.3	9.5
>¥8 million	210 (5.3)	4.4	5.6	12.4	8.7
Unknown	555 (14.1)	14.5	14.1	13.0	11.2
Chronic disease					
None	2871 (73.1)	74.0	70.8	73.9	73.1
Present	1059 (26.9)	26.0	29.2	26.1	26.9
Industrial classification					
Primary industry	305 (7.8)	8.7	7.4	0.6	5.0
Secondary Industry	1097 (27.9)	28.0	26.7	34.2	28.5
Tertiary industry	2528 (64.3)	63.3	65.9	65.2	66.5
Occupational classification					
White‐collar worker	1496 (38.1)	35.5	41.9	41.0	43.4
Blue‐collar worker	2434 (61.9)	64.5	58.1	59.0	56.6
Work shifts					
Day shift	3155 (80.3)	79.6	81.8	75.2	83.1
Both day and night shifts	87 (2.2)	2.2	2.2	1.9	2.1
Night shift	489 (12.4)	13.1	10.4	16.8	12.0
Flexes, etc.	199 (5.1)	5.0	5.6	6.2	2.9
Number of days of dental visits					
During working hours	Mean ± SD	0.14 ± 1.18	0.57 ± 2.67	0.21 ± 1.20	0.32 ± 1.29
Outside working hours	Mean ± SD	1.61 ± 8.61	4.60 ± 6.19	1.66 ± 4.35	2.41 ± 4.47
During and outside working hours	Mean ± SD	1.75 ± 8.74	5.17 ± 6.76	1.87 ± 4.70	2.73 ± 4.74

Table 2 shows the association found between each variable and absenteeism using linear regression analysis. After covariate adjustment, those who did not receive dental check‐ups (coefficient −0.43; 95% CI, −0.59 to −0.27), those who received check‐ups at the workplace (coefficient −0.35; 95% CI, −0.58 to −0.12), and those who received dental check‐ups at both the dental clinic and at the workplace (coefficient −0.23; 95% CI, −0.45 to −0.01) had significantly fewer days of absence due to dental clinic visits than those who received dental check‐ups at the dental clinic.

**TABLE 2:** Absolute difference in the number of days of dental visits during working hours based on the place of the dental check‐ups and confounders analyzed using linear regression analysis (*n* = 3930).

	Number of days of dental visits during working hours		Univariable model	Fully adjusted model
	Mean	SD	Coefficient (95% CI)	Coefficient (95% CI)
Total	0.28	1.75		
Place of dental check‐ups				
Not had the check‐ups	0.14	1.18	−0.43 (−0.59;−0.26)*	−0.43 (−0.59;−0.27)*
At the dental clinic	0.57	2.67	ref	ref
At the workplace	0.21	1.20	−0.36 (−0.60;−0.12)*	−0.35 (−0.58;−0.12)*
At both the dental clinic and the workplace	0.32	1.29	−0.25 (−0.48;−0.03)*	−0.23 (−0.45;−0.01)*
Self−reported oral health				
Excellent	0.31	1.85	ref	ref
Good	0.19	1.25	−0.12 (−0.34;0.10)	−0.13 (−0.35;0.09)
Fair	0.26	1.76	−0.06 (−0.28;0.16)	−0.07 (−0.29;0.16)
Poor	0.39	2.19	0.08 (−0.18;0.33)	0.01 (−0.25;0.27)
Very Poor	0.35	1.41	0.03 (−0.28;0.34)	−0.14 (−0.49;0.20)
Number of teeth				
0–14	0.77	2.75	0.53 (0.12;0.93)*	0.44 (0.05;0.82)*
15–19	0.58	2.61	0.33 (0.02;0.65)*	0.25 (−0.06;0.56)
20–23	0.21	1.41	−0.03 (−0.15;0.09)	−0.03 (−0.15;0.09)
24–27	0.21	1.69	−0.04 (−0.17;0.10)	−0.06 (−0.19;0.08)
28–32	0.25	1.63	ref	ref
Gum bleeding				
Always	0.50	2.13	0.26 (−0.07;0.60)	0.24 (−0.13;0.61)
Occasionally	0.32	1.72	0.08 (−0.03;0.19)	0.08 (−0.04;0.20)
None	0.23	1.75	ref	ref
Age (years)				
20–29	0.16	0.84	ref	ref
30–39	0.26	1.69	0.10 (−0.02;0.23)	0.08 (−0.05;0.20)
40–49	0.22	1.25	0.07 (−0.04;0.17)	0.01 (−0.11;0.12)
50–59	0.32	1.93	0.17 (0.02;0.31)*	0.06 (−0.10;0.21)
60–65	0.54	3.01	0.38 (0.09;0.67)*	0.21 (−0.07;0.49)
Gender				
Male	0.28	1.74	0.00 (−0.11;0.11)	−0.01 (−0.14;0.12)
Female	0.28	1.77	ref	ref
Education (graduation)				
High school	0.26	1.60	−0.01 (−0.12;0.11)	−0.05 (−0.18;0.07)
Vocational school, Junior college	0.35	2.22	0.08 (−0.08;0.25)	0.07 (−0.09;0.24)
University, Master's program, Doctoral program	0.26	1.61	ref	ref
Personal income				
<¥2 million	0.38	1.68	0.20 (0.01;0.38)*	0.18 (−0.06;0.42)
¥2–4 million	0.26	1.90	0.08 (−0.08;0.24)	0.10 (−0.09;0.29)
¥4–6 million	0.28	1.82	0.10 (−0.08;0.28)	0.12 (−0.07;0.32)
¥6–8 million	0.35	1.74	0.17 (−0.06;0.41)	0.18 (−0.06;0.42)
>¥8 million	0.18	0.95	ref	ref
Unknown	0.22	1.54	0.04 (−0.14;0.23)	0.09 (−0.11;0.30)
Chronic disease				
None	0.18	1.25	ref	ref
Present	0.53	2.67	0.35 (0.18;0.52)*	0.26 (0.12;0.41)*
Industrial classification				
Primary industry	0.54	3.01	ref	ref
Secondary Industry	0.30	1.76	−0.24 (−0.59;0.11)	−0.15 (−0.48;0.19)
Tertiary industry	0.24	1.53	−0.30 (−0.65;0.04)	−0.22 (−0.55;0.11)
Occupational classification				
White‐collar worker	0.23	1.42	ref	ref
Blue‐collar worker	0.31	1.93	0.08 (−0.03;0.18)	0.05 (−0.06;0.15)
Work shifts				
Day shift	0.26	1.68	ref	ref
Both day and night shifts	0.47	2.66	0.21 (−0.35;0.77)	0.17 (−0.37;0.72)
Night shift	0.25	1.76	−0.01 (−0.18;0.16)	0.03 (−0.15;0.20)
Flexes, etc.	0.52	2.36	0.26 (−0.07;0.59)	0.21 (−0.12;0.53)

* *P* < 0.05.

## 4 DISCUSSION

This is the first study to examine the association between the place of dental check‐ups and absenteeism among Japanese workers. Compared to people who received dental check‐ups at dental clinic privately, those who received dental check‐ups at the workplace had significantly fewer days of absence due to dental clinic visits. Therefore, conducting dental check‐ups as a part of workplace health promotion could reduce absenteeism due to visits to the dental clinic compared with undergoing dental check‐ups at the dental clinic.

In this study, we examined the association between the place of dental check‐ups and absenteeism among Japanese workers due to dental visits. To the best of our knowledge, previous studies in this direction has not been conducted yet; however, some studies have reported that workplace health promotion programs reduce presenteeism and improve productivity.^18^, ^19^ In our study, reduction in absenteeism due to dental check‐ups conducted at workplace may be attributed to the fact that such check‐ups might provide an opportunity for performing regular oral screening and providing health education. Therefore, workplace dental check‐ups were considered to function as a workplace health promotion program.

This study implicates the usefulness of dental check‐ups as health promotion in the workplace. People with acute dental problems are more likely to be absent from work than those without, and absenteeism is especially high when they have oral cavity pain.^20^ People with poor oral health are generally more likely to experience acute dental symptoms. Regular dental check‐ups provide the opportunity for early detection and treatment of dental diseases, which improves the oral health of the patients.^21^ Dental check‐ups at the workplace may also increase the likelihood of early detection and treatment of dental diseases and reduce acute dental problems. Moreover, among those who received dental check‐ups at the dental clinic, it is assumed that the group of those who received regular dental check‐ups was mixed with those who visited the dental clinic because of acute symptoms and then received dental check‐ups at that time. Oral health is often neglected in universal health coverage.^22^, ^23^ Dental check‐ups at the workplace could improve dental health care coverage among the working population. We found that high proportion of male participants and participants with low levels of education and income tended to underwent no dental check‐ups. Further, participants who did not receive dental check‐ups tended to have a poor oral health status, although their absenteeism was low. If such workers are given the opportunity to receive regular dental check‐ups at their workplace, their oral health status may improve.

This study has several limitations. For instance, this study did not distinguish between mandatory special dental check‐ups and general workplace dental check‐ups. Special dental check‐ups mandated by law are conducted once every 6 months or less in Japan for workers working with acids or other toxic substances. However, the proportion of workplaces handling acids and other toxic substances that were obligated to conduct special dental check‐ups was 1.6%, and only 31.5% of these workplaces had conducted special dental check‐ups.^24^ Therefore, the percentage of patients who receive special dental check‐ups is sufficiently small. As another limitation, temporal association cannot be determined since this is a cross‐sectional study. A longitudinal study design would be necessary to investigate the place of dental check‐ups and absenteeism. However, reverse causation, dental check‐ups increase due to absence due to dental visits is perhaps theoretically unrealistic.

## 5 CONCLUSIONS

Workers who received dental check‐ups at the workplace were associated with less absenteeism due to dental visits than workers who received dental check‐ups at the dental clinic.

## AUTHOR CONTRIBUTIONS

Sayo Masuko and Jun Aida conceived the ideas; Takashi Zaitsu and Akiko Oshiro collected the data; Sayo Masuko and Jun Aida analyzed the data; and Sayo Masuko, Miho Ishimaru and Jun Aida led the writing.

## CONFLICT OF INTEREST STATEMENT

Authors declare no Conflict of Interests for this article.

## DISCLOSURE

*Approval of the Research Protocol*: The Ethical Review Committee of the School of Dentistry, Tokyo Medical and Dental University (Approval No. D2015‐526) approved this study. *Informed Consent*: Participants provided written informed consent upon registration. *Registry and the Registration No. of the Study/Trial*: N/A. *Animal Studies*: N/A.

## Data Availability

The data that support the findings of this study are available from the corresponding author upon reasonable request.
